# The Invisible Cliff: Abrupt Imposition of Malthusian Equilibrium in a Natural-Fertility, Agrarian Society

**DOI:** 10.1371/journal.pone.0087541

**Published:** 2014-01-31

**Authors:** Cedric Puleston, Shripad Tuljapurkar, Bruce Winterhalder

**Affiliations:** 1 Department of Anthropology, University of California Davis, Davis, California, United States of America; 2 Department of Biology, Stanford University, Stanford, California, United States of America; University of Washington, United States of America

## Abstract

Analysis of a natural fertility agrarian society with a multi-variate model of population ecology isolates three distinct phases of population growth following settlement of a new habitat: (1) a sometimes lengthy *copial* phase of surplus food production and constant vital rates; (2) a brief *transition* phase in which food shortages rapidly cause increased mortality and lessened fertility; and (3) a *Malthusian* phase of indefinite length in which vital rates and quality of life are depressed, sometimes strikingly so. Copial phase duration declines with increases in the size of the founding group, maximum life expectancy and fertility; it increases with habitat area and yield per hectare; and, it is unaffected by the sensitivity of vital rates to hunger. Transition phase duration is unaffected by size of founding population and area of settlement; it declines with yield, life expectancy, fertility and the sensitivity of vital rates to hunger. We characterize the transition phase as the *Malthusian transition interval (MTI)*, in order to highlight how little time populations generally have to adjust. Under food-limited density dependence, the copial phase passes quickly to an equilibrium of grim Malthusian constraints, in the manner of a runner dashing over an invisible cliff. The three-phase pattern diverges from widely held intuitions based on standard Lotka-Verhulst approaches to population regulation, with implications for the analysis of socio-cultural evolution, agricultural intensification, bioarchaeological interpretation of food stress in prehistoric societies, and state-level collapse.

## Introduction

Malthus [1], Boserup [2], Wood [3], Lee [4],[5] and others have given us riches of partial insights and unresolved puzzles on the topics of population, environment, economics and social evolution. Despite an extensive intellectual history and acknowledged importance, our methods for analyzing population and ecology are not well developed. We commonly fall back on the insights of a few pioneers and analytical models of too few parameters. Many of us have formed our basic intuitions from conceptual bases not much advanced over those developed in the 18^th^ and early 19^th^ century by Verhulst (1804–1849), Lotka (1880–1949), and Volterra (1860–1940) (see [6]). Our models of these evolutionary and historical processes often are built from too few variables to accurately represent their dynamics [7],[8],[9]. For example, these approaches begin by defining carrying capacity as a parameter instead of treating it as a variable dependent on features of the population and its environment. This makes it difficult to analyze realistic population dynamics and regulation. Holling [10] and others have made headway into developing simple yet powerful models of forager behavior and predator-prey dynamics in animal populations, but these are only distantly applicable to human agriculturalists, and have nothing to say about the links between food, labor, social and economic behavior and demography.

These complex interactions were considered in schematic form in a classic work by Wood [3] and later incorporated into a modeling framework called “food-limited demography,” developed in Lee and Tuljapurkar [11], Puleston and Tuljapurkar [12] and Lee *et al.* [13]. We extend it here to examine the nature and timing of the trajectories of food-limited agricultural populations. We examine the interplay between the size and demographic characteristics of a population and the production and consumption of its food supply. The variables and dynamics we seek to understand rest at the heart of problems from the prehistoric origins of agriculture and the state, to contemporary issues of population and environmental degradation. The framework iteratively assesses the effects of (a) age-sex distribution and population size on a society's labor availability and food requirements; (b) environmental factors affecting yield of resources, given labor; (c) consumption requirements of the population, given its size and age composition; and, (d) the effect of food availability on vital rates of fertility and mortality. From these linked interactions flows a revised schedule of fertility and mortality, leading to a new age distribution and population size, completing the logical circuit of the model. How much the population grows or shrinks depends on how much food it can grow using the available labor pool, technology and skills, and the agrarian landscape it occupies. A well-fed population can grow rapidly, but a hungry one will decline from elevated risk of death and lowered fertility.

Here we apply the model to examine the developmental course of an agrarian society, from initial settlement to equilibrium, as a means of illuminating relationships between population history, environment and density dependent controls. This paper anticipates a second [14] in which we explore the interaction of agrarian production, peasant livelihood and the state-level extraction of revenue in the form of goods or labor.

We envision a population colonizing an empty habitat and show that the resulting population trajectory is characterized by three stages: (i) a relatively long period of food abundance (*copial phase*), followed by (ii) a generally very short *transition phase*, to a (iii) *Malthusian phase* representing a stable equilibrium of indefinite length. We estimate the relative length of these phases as a function of basic environmental and demographic properties. Of special interest is the transition phase (ii), the period initiated by the first impacts of food shortfalls on vital rates. We define the term *Malthusian transition interval (MTI)* to characterize the span between the first experience of hunger and when the population effectively reaches its Malthusian equilibrium. This period represents the interval in which the effects of population pressure have become obvious, but in which there still may be room to make behavioral adaptations to forestall a density-dependent equilibrium. We bound the end of this interval at the arbitrary point when the population's doubling time has slowed to 1000 years. The population is effectively stationary at this point, even though it will continue to approach equilibrium for an infinite span. We show that the *MTI* typically is quite short and, counter-intuitively, that it is shorter the better are the copial phase circumstances of the population. Higher environmental yield, longer lifespan, lower background mortality and higher fertility all act to impose a more abrupt, shorter and, we presume for the populations experiencing them, more painful passage to the full effects of Malthusian food limitations. Like a runner moving quickly over flat ground, the faster the pace, the less time to react when the road begins to fall away beneath her.

We find population ecology dynamics that differ in important ways from those generated by Lotka-Verhulst approaches. Citing examples from Maya prehistory, we show how the model can be used to evaluate hypotheses to the effect that labor shortages, peasant revolt, or cyclic structural instabilities contributed to state level collapse. We likewise show how bioarchaeologists who adopt Lotka-Verhulst expectations are likely to make incorrect empirical inferences about the impact of population on societal development.

## Model

### The Conceptual Model

We focus on food- and space-limited population dynamics in a constant environment, and on colonization of an empty habitat. Our results would pertain as well to populations recovering from severe declines in their numbers, or to those suddenly released from extant density dependent constraints by environmental changes or technological innovations that significantly elevate subsistence production.

**Figure 1:**
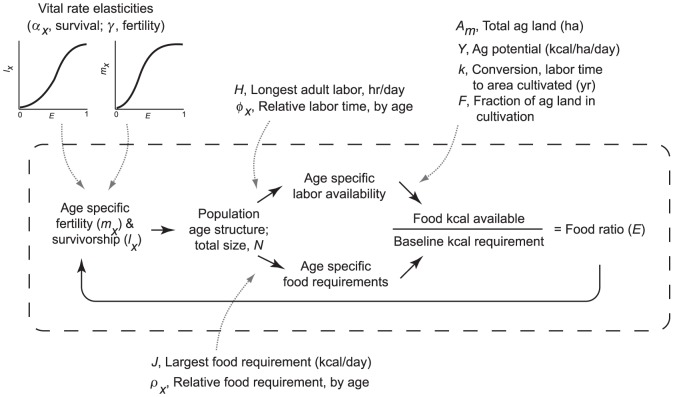
Population Ecology in an Agrarian Society. Components within the dashed rectangle make up the model itself; solid arrows depict the causal relationships that link them. Model parameters, those that must be set and can be manipulated for analytical purposes, are shown outside the dashed box. Dotted arrows show their point of operation in the logical structure of the model. Time is indexed implicitly in all model terms; *x* indexes age.

The model is depicted in Figure 1; parameters are defined, assigned ranges and units and given brief descriptions in Table 1. We work through the conceptual and mathematical relationships of the model, and then turn attention to the adjustable parameters that allow us to make analytical and interpretive use of it. Model mechanics are represented by the components within the dashed rectangle of Figure 1.

**Table 1:** Model components and parameters.

Parameter	Interpretation	Baseline value & unit	Socio-environmental features represented in this parameter
*Y*	yield/area	21,000 kcal/ha/day	Edaphic conditions, climate, cultigens, technology
*H*	longest age-specific agricultural work day	5 hr/day/ind (10 hr/day/worker)	Climate, physiological stress, socio-economic factors, socio-cultural belief and practice concerning agrarian labor
*k*	area worked/hr	0.0944 ha-day/worker/hr	Topography, technology, energy subsidies
*φ_x_*	proportion of hours, *H*, worked by age	0≤*φ_x_*≤1; *φ*_0_ = 0.738	Age-specific physiological and skill-related constraints on work; socio-cultural belief and practice related to labor
*m_x_*_(0)_	maximum, age-specific fertility, *E*≥1	daughters born to a woman from age *x* to *x*+1	Life history context; socio-cultural belief and practice
*p_x_*_(0)_	maximum, age-specific survival, *E*≥1	probability of survival from age *x* to *x*+1	Physiological and epidemiological environment; socio-cultural belief and practice
*J*	Maximum, age-specific kcal requirement	2785 kcal/day	Body mass, climatic conditions, metabolic costs of maintenance and reproduction
*ρ_x_*	Proportion of consumption, *J*, by age	0≤*ρ_x_*≤; *ρ*_0_ = 0.832	Age-specific nutritional needs; socio-cultural belief and practice related to food distribution
*A_m_*	Arable land available	1000 ha	
*F*	Fraction of *A_m_* in cultivation	0≤*F*≤1	A function of population density and interference competition, decreasing returns to marginal land, etc.
*α_x_*	Elasticity at *E* = 1, at age *x*, for survival rate, *p_x_*	0.00279≤*α_x_*≤0.156; *α_25_* = 0.00464 at *E* = 1	Climate, other environmental factors affecting age-specific survival under conditions of food shortfalls from optimum; physical environment, disease, cultural practices, technology
*γ*	Elasticity at *E* = 1, at any age for fertility, *m_x_*	*γ* = 0.135 at *E* = 1	Any socio-environmental factor affecting age-specific fertility under conditions of food shortfalls from optimum physical environment, disease, cultural practices, technology
*E*	Food ratio	*E_t_*_ = 1_ = 2.99; 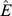 = 0.6683	
*r*, *b*, *d*	per capita reproductive (*r*), birth (*b*) and death (*d*) rate	*r*_0_ = 0.0176; *b*_0_ = 0.0369; *d*_0_ = 0.0192	
*TFR_d_*	Total fertility rate, in daughters	*TFR_d_* = 2.44	
*e*_0_	Life expectancy, from birth (age 0)	*e*_0_ = 45 yr	
*N_t_*	Population size at time *t*		

The model tracks the age structure and size of the population (“Population age structure; total size, *N*”). We conceptualize all individuals as female and make adjustments to their fertility schedule in such a ways as to include an equal number of phantom males. The number of individuals in each annual age class determines “Age specific labor availability” and “Age specific food requirements.” Available labor determines total production of food according the specific environmental and socio-economic parameters that characterize the production system. Production is then summed as “Food kcal available.” Age specific food requirements likewise are summed, generating the “Baseline kcal requirement,” baseline in the sense that it is the minimum kilocalories required for all individuals in the population to achieve their optimal vital rates.

The “Food ratio (*E*)” characterizes our approach as one of food-limited demography. If the age-specific labor supply is large enough and environmental conditions accommodate sufficient production, then more food will be available from subsistence production than is required for the population to meet its baseline requirements and *E*≥1. If not, *E*<1, and the population exists in a state of suboptimal diet measured as kcal deprivation relative to the baseline. More simply, *E*<1 represents a state of hunger.

The operational effect of this condition is shown in the component “Age-specific fertility (*m_x_*) & survivorship (*l_x_*).” If *E*≥1, then fertility and survivorship are at their baseline maxima. As *E* drops below 1, food deprivation depresses fertility and elevates mortality. A gamma probability density function represents the response of fertility (*γ*) and age-specific survival (*α_x_*) to shortfalls. These curves are parameterized from empirical data (discussed in [11]) showing that sensitivity to kcal shortfalls and hunger is initially small but becomes quite large as food availability falls toward 50% of baseline level.

Continuing the cycle, age-specific fertility and mortality then have their actuarial consequences in an updated “Population age structure; total size, *N*,” completing an iteration of the model. The time index advances one step, a new year begins.

For ease of comprehension we have described the model as a loop of causal interactions. In fact, it is represented and iterated or solved as a family of projection matrices (see [15]), with linear algebra and occasionally the software environment of MatLab® doing the heavy work.

The components outside of the dashed rectangle are the system parameters. Where these require a specific value we have chosen figures representative of prehistoric subsistence populations engaged in agriculture (Table 1 and see [11]). The exact value chosen is less important than the opportunity to examine the effect of that parameter varied over a range of potentially realistic values. Thus, rather than asking “What is the equilibrium population density of humans if yield (*γ*) = 21,000 kcal/ha/day?,” we might ask, “What is the effect of doubling yield on population density or life expectancy?” We use the model to make *structural* predictions [16] of the form, “If *x* gets larger, what is the direction and relative magnitude of the effect on *y*?”

*H* (Figure 1) is the number of hours of productive subsistence work per day by the age class or classes with the greatest daily labor investment in producing food. The parameter *φ_x_* determines the relative work investment by any age class *x*, scaled to 0≤*φ_x_*≤1. By definition, *φ_x_* = 1 for individuals working *H* hours; *φ_x_* = 0 for complete dependents such as infants and the very elderly. Together, *H* and *φ_x_* give us control over the age specific subsistence work effort of the population (“Age-specific labor availability”). We define *φ*_0_ as the sum over all age classes of each *φ_x_* times the fraction of the copial population in that class. This tells us how many “full-time” equivalent workers there are per person and, as such, is a measure of the dependency ratio.

The “Age-specific food requirements” (kcals) are handled similarly. *J* is the requirement of the age class or classes with the greatest food needs and *ρ_x_* scales the requirement for all other age classes, 0≤*ρ_x_*≤1. We determine *ρ_x_* according to estimates from representative modern populations; it is independent of the level of agricultural labor assigned an age class, *φ_x_*. *ρ*_0_ measures the equivalent number of most energy costly individuals per capita in the copial phase. Food is allocated across age classes in equal proportion to need.

The next group of parameters sets the environmental conditions of production. *A_m_* gives the total area (ha) of arable land available. We assume it to be of uniform quality with an average annual food yield (*γ*) measured in kcal/ha/day. *k* is a constant that converts labor time into area cultivated.

*F* captures the difficulty of putting into production increasingly marginal plots as the population grows. At its simplest, *F* would be a straight line of positive slope, each new farmer bringing into cultivation the same increment of land until the habitat was fully allocated and more agriculturalists do not lead to an increase in food supply. We assume that interference competition results in a steadily decreasing efficiency of agricultural expansion by employing a continuous exponential function that is nearly linear when the population is small. Concave downward, its slope approaches an asymptote of *F* = 1 as *N* becomes large.

Finally, we use a gamma probability density function (*pdf*) (“Vital rate elasticities”) to determine how vital rates of age-specific survival and age-specific fertility respond to food shortfalls. The sigmoid form suggests that modest kcal shortfalls of *E*<1 have a minimal effect, but that more significant food deprivation (0.5<*E*<0.8) has a rapidly accelerating impact on survival and fertility. The slope of the gamma pdf determines the local *elasticity* or responsiveness of survival and fertility to food shortages (see [15]). The shape of the gamma pdfs has been calibrated to empirical observations [11],[17]. These elasticities are bio-cultural in that they are determined by socio-economic factors and choice as well as by hormonal and metabolic responses to food deprivation.

Linkages between nutrition, fertility and mortality are complex and the relationships we employ may not hold in all cases, particularly as they include behavioral components expected to vary across societies. We find that the broad conclusions of the analysis remain robust even if fertility rates are entirely unresponsive to food availability. In this case equilibrium is achieved by elevated mortality alone. Of course because fertility is a parameter in our analytical expressions, it is possible to consider the results of varying the sensitivity of fertility across a wider range than we have done here.

The food-limited framework has advantages over the Lotka-Verhulst approach. Most significant, “carrying capacity” need not be set *a priori*. Instead, the number of individuals at equilibrium is a function of the interaction between labor, environment, food availability and vital rates. By representing population dynamics in this way, we can examine their dependence on factors not present in the logistic framework. Similar advantages arise from deriving growth rate from underlying processes of fertility and mortality. A central variable of the model, the food ratio, drives vital demographic rates but also serves as a measure of human welfare, for which there is no analog in Lotka-Verhulst. Finally, the food-limited approach allows the consideration of any number of novel socio-economic features, such as the effects of the method and severity of taxation [14], innovation that increases either yield or worker efficiency [12], and storage [18].

The most important cost of this approach: its complexity means we often must forsake exact analytical solutions for approximations and simulation. Another is that the large number of parameters require significant effort to tailor the approach to new scenarios. It could also be argued that that the model is not complex enough. It relies on numerous simplifying assumptions, and factors that do not appear may in fact be quite important. Our goal is to better understand the interactions of the features that are included, not to discount any that might be missing. We prize tractability for the analytical insights it affords us, even at the cost of some realism.

### The Model as Mathematics

We solve for copial phase dynamics analytically. To examine dynamics in the remaining phases we iterate the population forward one year at a time, beginning with a small founder group and baseline rates of survival and fertility, indicating that enough food is available to avoid discounting vital rates due to hunger. The food-dependent vital rates are converted to a Leslie matrix to calculate the next year's population from the current population vector. This is equivalent to multiplying the age-specific probabilities of survival by the appropriate numbers of people in each age group going forward. Newborns are created by doing the same with the age-specific fertility rates.

In some cases we calculate an equilibrium state from initial parameters, regardless of the pathway of transitory states, using the method of comparative statics. This entails solving for the value of *E* at which the population equilibrates. This must be determined numerically. The point at which the population just replaces itself each year is the value of *E* for which the dominant eigenvalue of the food-dependent Leslie matrix is 1. The associated eigenvector is proportional to the equilibrium population structure. Sections 2 and 3 of File S1 give more details.

## Results

### Growth to Equilibrium and Its Consequences

#### A Standard Temporal Pathway

**Figure 2:**
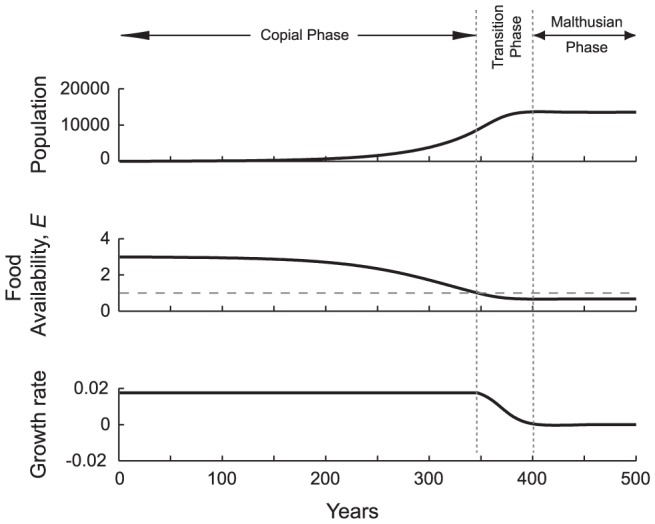
Growth in a Space-Limited Agrarian Population. From top to bottom the graphs show concordant changes in population size (*N*), food availability (*E*) and population growth rate (*r*). At time zero a group of 20 people (*N*_0_ = 20) colonize a productive but space- and resource-limited agricultural landscape. High food availability (E>1) and an open frontier for settlement allow the population to grow at its maximum unconstrained rate for 346 years, when it becomes sufficiently large that the environment is filled and can no longer provide the amount of food energy optimal for baseline vital rates. As food availability falls below *E* = 1, convergence between per capita fertility and mortality rates causes a decline in growth rate (*r*) and the population asymptotically approaches an equilibrium. We describe this time course in terms of a *copial* phase, in which the population is demographically unconstrained and growing at this maximum rate; a short *transitional* phase, in which fertility declines and mortality increases; and, a *Malthusian* phase in which fertility matches mortality and growth ceases. Model parameters initially set to: *TFR_d_* (measured in daughters) = 2.44; average lifespan *e*_0_ = 45 years; agricultural area *A_m_* = 1000 ha; yield *Y* = 21,000 Kcal/ha/day; youths start work at age 10 and adults end working life at 65.

In Figure 2 a population of 20 settles in a rich but unoccupied landscape (*A_m_* = 1000 ha; *Y* = 21,000 kcal/ha/day). Their total fertility rate (daughters only) is *TFR_d_* = 2.44, and their lifespan is *e*_0_ = 45 years. At the time of arrival the food ratio, *E*, is just below 3. The population can produce three times the food required to optimize its vital rates. Consequently, the growth rate is at its baseline maximum, *r* = 0.0176. From this founding event, the population grows and the food ratio declines. For 346 years the birth rate remains high and undiminishing, the death rate low and not increasing. At year 347 the food ratio declines below *E* = 1 and the growth rate plummets as vital rates react to food shortfalls; total population growth decelerates. Forty years later, suboptimal food availability drives the net growth rate *r* approximately to zero by substantially degrading survival and fertility. All three variables of Figure 2 settle asymptotically on the value that they will maintain indefinitely. There are 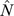 = 13,509 individuals, consuming 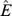 = 0.668 of their optimal diet and achieving an average expected lifespan 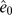 = 30.0 yr.

We divide this temporal path into three phases: (1) a *copial phase*, the period of potential surplus production between initial settlement and the point at which suboptimal food intake (hunger) is first experienced (i.e., *E*<1); (2) a *transition phase*, which represents a period of increasing food-limited constraint on reproduction and survival; and (3) an *equilibrium or Malthusian phase*, in which food-limitations have caused rates of birth and death to converge, stabilizing population size. Separation between phases (1) and (2) is precise at *E* = 1. For the purposes of calculating the *MTI* we set the dividing line between phases (2) and (3) at a doubling time of 1000 years, the point at which growth virtually has stopped.

#### Malthusian Transition Interval (MTI)

Much of our analysis concerns the effect of environmental and social factors on the relative duration of the first two of these temporal phases, distinguished as a long and bucolic period of relative plenty and a much shorter period of rapid adjustment to a final period of severe Malthusian constraints based in hunger. To focus this effort, we introduce the concept of *Malthusian transition interval (MTI)* to characterize the transition phase.

Consider this scenario. A small population arrives as first colonists in a region with well-defined geographical boundaries (e.g., an island or valley). The climate, the state of its soil, and the technology, skills and cultivars available to the population are propitious. The founding group begins with a potential surplus of food (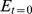
>1). The population grows exponentially at a constant rate (*r*_0_) determined by its baseline fertility and mortality. Its frontier of unused agricultural land diminishes. Interference competition begins to constrain per capita access to land, captured by the flattening slope of *F* (the fraction of arable land that is in cultivation). Surplus declines to and then drops below *E* = 1. As per capita rates of death and birth converge due to food shortfalls, population growth stops.

**Figure 3:**
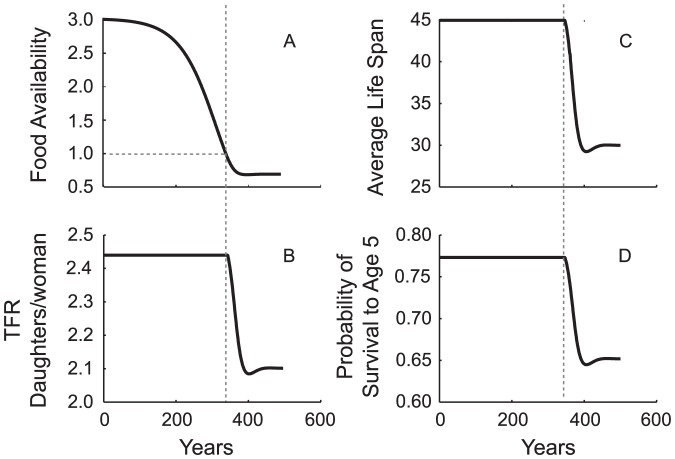
Malthusian transition interval (*MTI*) and Quality of Life Demographic Variables. (A) Food availability declines from *E* = 2.99, to *E* = 1 at year 347. It stabilizes at *E* = 0.668 approximately 51 years later. Coincident with the beginning of the transition phase: (B) total fertility rate (*TFR_d_*) declines sharply from 2.44 to replacement, 2.10; (C) average life span falls from *e*_0_ = 45 to 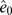 = 30.0 years; and (D) the probability of a youth surviving to age five falls from 0.773 to 0.652. Although the production surplus declines continuously and gradually, Malthusian constraints impose themselves abruptly as variables representing demographic quality of life plummet.

The start of the transition phase has a sudden and palpable impact on human quality of life (Figure 3). Life expectancy is diminished and infant-youth mortality is elevated. Total fertility rate falls to replacement. These changes likely would be unexpected and they happen quickly. Total fertility rate, average life span and probability of survival to age five are high and constant throughout the copial phase, but once *E* drops below 1 they plummet toward their equilibrium values. In model analyses with realistic parameters the copial phase typically lasts between 300 and 400 years (12–16, 25-year generations). The transitional phase and *MTI* are much shorter, often less than 50–60 years (two-three generations).

*MTI* highlights the implications of these two time frames. Density dependence strikes with little warning. It sharply and quickly curtails human quality of life by elevating hunger and infant mortality, and by lowering life expectancy and fertility. Between the initial signal of a problem and its full impact, the opportunity for adaptation to avert the full force of the Malthusian outcome is short. We believe this has implications for the effects of population ecology on socio-economic history.

### Factors Affecting Phase Duration

The effects of environment, work, consumption, and demography on the duration of the copial and transitional phases of population growth are not easy to predict. Generally this transition occurs rapidly, but in a narrow range of specific circumstances it can be prolonged. We examine the consequences of altering initial colony size (*N_t_*_ = 0_), the area of the habitat being settled *A_m_*, environmental yield (*Y*), baseline life expectancy (*e*_0_), total fertility rate (*TFR_d_*), and the elasticity of fertility and age-specific survival at *E* = 1 (*γ*, *α_x_*) (see also supplemental materials in File S1).

#### Founding Population Size

**Figure 4:**
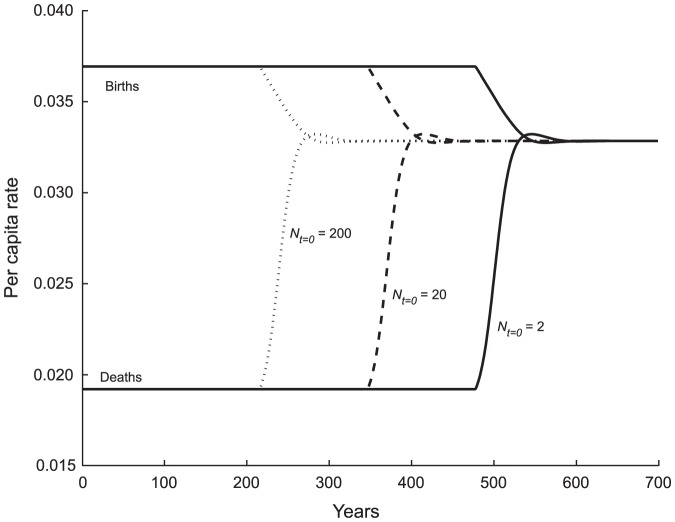
Effect of Settlement Population Size *N*_0_ on the Time Course of Vital Rates. Increasing the size of the founding group by factors of 10, from 2 to 20 to 200, decreases the length of the copial phase by a constant 131 years each step, a log-linear relationship. During the copial phase per capita births and death rates, hence the reproductive rate, *r*, are unchanging. The transition phase in which birth and death rates converge (at *b* = *d* = 0.033) arrives sooner with larger settlement, but it otherwise is of the same form and duration for each scenario. At Malthusian equilibrium, the population numbers 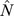 = 13,509 individuals, who have a life expectancy of 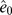 = 30.0 yrs; the food ratio is 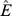 = 0.668.

A larger initial settlement group shortens the copial phase; however, the duration of the transitional phase or *MTI* is unaffected. We demonstrate this and subsequent results in two formats. Figure 4 shows it in terms of the vital rates that drive population growth; Table S1a (File S1; Section 1) shows the duration of the copial phase declines with increasing size of the founding group, whereas the duration of the transitional phase is constant over *N*_0_. During the copial phase a constant per capita birth rate exceeds a constant per capita death rate by a wide margin (Figure 4); *r* is relatively large. As *E* drops below 1, initiating the transitional phase, these rates begin rapidly to converge. They overshoot slightly and then settle on the equalized value that determines replacement without growth, the Malthusian equilibrium. Per capita birth rate declines from 0.037 to 0.033 (a drop of 11%), whereas per capita death rate climbs from 0.019 to 0.033 (an increase of 71%). Our parameterization of vital rate elasticities often results in mortality being more responsive to food deprivation than is fertility. 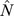 = 13,509, life expectancy 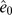 has fallen from 45.0 to 30.0, and 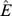 = 0.668, indicating that at equilibrium the population is consuming 67% of its demographically optimal diet.

#### Area of Habitat Available for Settlement

**Figure 5:**
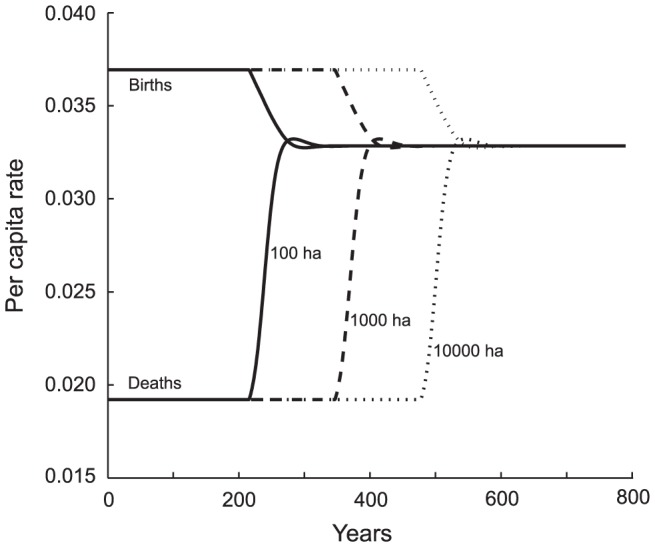
Effect of Agricultural Area (*A*_m_) on the Time Course of Vital Rates. Increasing the maximum area available for agricultural in-fill (*A*_m_) by factors of 10, from 100 to 1000 to 10,000 ha, increases the length of the copial phase by a constant 131 years each increment, a log-linear relationship. Habitat area has no effect on transition phase length; the passage time from E = 1 to the neighborhood of the Malthusian equilibrium is identical in all cases. At equilibrium, food availability (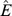 = 0.668) and lifespan (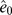 = 30) are unaffected by area. By contrast, total population size increases proportionally: 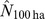 = 1351; 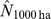 = 13,509; 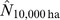 = 135,090.

As the available habitat grows larger, the copial phase increases in duration. The duration of the relatively short transitional phase is unaffected by area (Figure 5; File S1 Table S1b). Increasing the agricultural land available (*A_m_*) reduces the initial the density of farmers, lowering interference competition among them. Populations able to expand over more arable land will take longer to feel the density dependent effects of hunger and diminishing returns on labor. As a result, high marginal productivities are prolonged and the decline of surplus (*E*≥1) is slowed. *MTI* however is independent of the amount of arable land (File S1 Table S1b). The point at which *E*>1 is associated with a particular population *density* and the effect of density on the timing of transition phase approach to equilibrium is invariant across different sized habitats.

Population grows at a constant exponential rate in the copial phase, ensuring that the effect of agricultural area on in-fill is log-linear. For our parameter set, increasing arable area by a factor of ten (from 100 ha to 1000 ha) adds 131 years to the copial phase. A second 10-fold increase adds another 131 years. The same log-linear effect is evident for founding group size (Figure 4).

#### Habitat Yield

**Figure 6:**
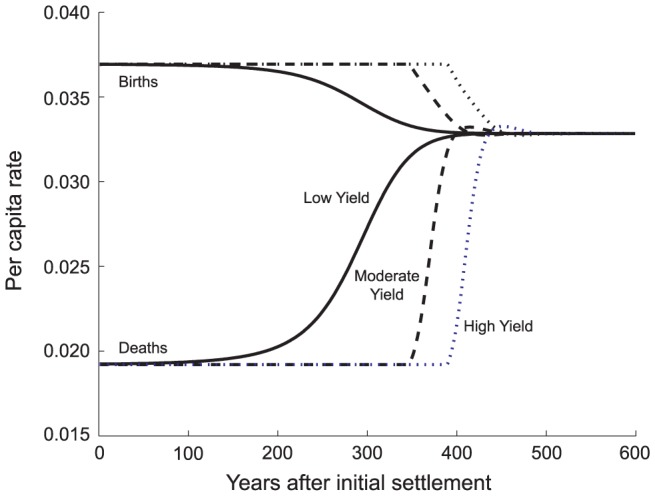
Effect of Yield (*Y*) on the Time Course of Vital Rates. The solid, low-yield, curves represent 7000 kcal/ha/day; the dashed, moderate yield curves a 3-fold increase to 21,000 kcal/ha/day; and, the dotted, high-yield curves another 3-fold increase to 63,000 kcal/ha/day. Because the low yield situation is below adequate kcal intake at settlement, there is no copial phase as fertility falls and mortality rises smoothly with further declines in food availability. The approach to equilibrium is slow, the transition phase extended. The moderate- and high-yield cases allow the population to grow at its maximum, constant rate until food availability drops below *E* = 1. They exhibit a lengthy copial phase. The moderate-yield population experiences hunger first, but in each case the approach to equilibrium is rapid once *E* has fallen below 1. At equilibrium, 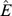 = 0.668 and 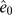 = 30.0 for each level of yield. Equilibrium population density, however, increases with yield; 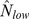 = 2799; 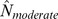 = 13,509; 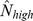 = 27,287.

Increasing the yield or productivity of the environment independent of its area lengthens the copial phase and it shortens the transitional phase (Figure 6; File S1 Table S1c). The extension of the copial phase is a result of the greater capacity of the system to produce a food surplus (i.e., a greater *E_t_*_ = 0_). Maximum growth rates can be sustained for a long period and the size, and hence density, of the population will be larger at the point that *E* first falls below 1. Thus even though the equilibrium population size increases approximately linearly with yield [12], the *MTI* is an expression of the exponential growth equation (see File S1 Equation S3.8). A richer yield per hectare increases the maximum value of *E*, and as a consequence, populations in high-yield environments pass through the transitional phase more quickly.

In a very low yield environment, a population experiences hunger early and at low density, with the result that it grows more slowly toward its equilibrium size. Settings of poor agricultural yields are unique in that *MTI* may be relatively long and may exceed the copial phase in duration (File S1 Table S1c).

These relationships are evident in vital rates (Figure 6). Low yielding environments may have no copial phase or a quite short one matched to a relatively long and gradual transitional phase witnessing the slow convergence of birth and death rates. Moderate to high yielding environments revert to the more common pattern in which there is a copial phase significantly longer than the transitional one and, although delayed, the transition happens with more typical abruptness. Intuition might suggest that adding arable land to a society's territory would have an effect similar to that of increasing the yield of a smaller area. This is not the case. While larger area (*A_m_*) and yield (*Y*) both increase copial phase duration, only the latter has an effect on the transitional phase. The analytical approximation of *MTI* (File S1 Equation S3.8) tracks the trajectory of population density, which is a function of yield, but not area.

Comparison of Figures 4, 5 and 6 shows that births and deaths equilibrate at the same value (0.033) for all treatments involving these three parameters (*N_t_*_ = 0_, *A_m_* & *Y*). Life expectancy at equilibrium likewise is the same across all cases, 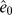 = 30.0, as is the hunger level, 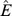 = 0.668. The size of the population at equilibrium does not change as a result of different settlement numbers (*N_t_*_ = 0_); however, it increases as a function of area (*A_m_*) and yield (*Y*). The Malthusian phase life history experience of individuals is unaffected by the size of the founding group, the area settled or the yield of the environment, parameters which affect the manner in which the equilibrium is approached but not its demographic or nutritional character. Among the other inferences possible, high yields do not improve quality of life at Malthusian equilibrium.

#### Background Mortality

**Figure 7:**
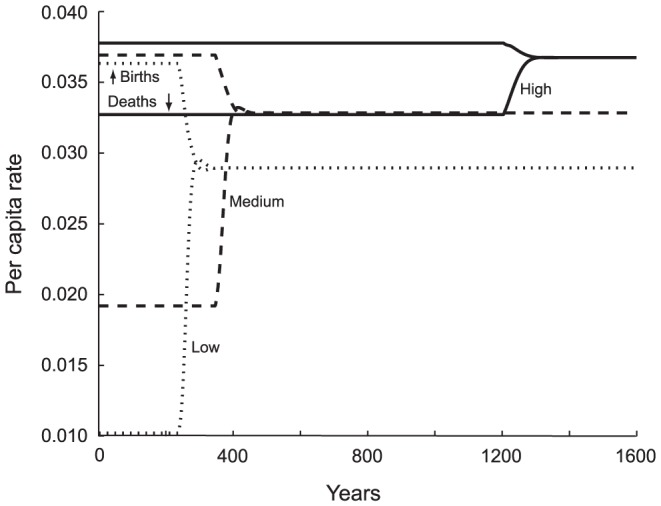
Effect of Background Mortality, Measured as Life Expectancy (*e*_0_), on the Time Course of Vital Rates. The solid lines represent high background mortality corresponding to a short life expectancy of *e*_0_ = 30 years. The dashed curves depict a medium mortality, baseline life expectancy of *e*_0_ = 45 years; the dotted lines low background mortality, baseline expected lifespan of *e*_0_ = 60 years. In the high-mortality environment, the maximum growth rate is small and the copial phase lasts a lengthy 1204 years. Mortality starts high and it changes very little at equilibrium (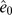 = 26.8) because equilibrium food availability is fairly high (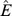 = 0.851). By contrast, the fast-growing, low-background-mortality population experiences a short copial phase of 237 years. Once it drops into hunger (*E* = 1) it approaches its Malthusian equilibrium very quickly, experiencing a large rise in mortality. At equilibrium, expected lifespan is longest in the most favorable environment (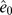 = 34.0, having however dropped from *e*_0_ = 60). Hunger is acute in this case. In numbers: High mortality, Harsh Env.: 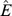 = 0.851; 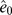 = 26.8; 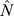 = 10,441. Moderate Mortality Env.: 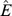 = 0.668; 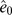 = 30.0; 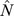 = 13,509. Low Mortality, Propitious Env.: 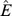 = 0.562; 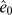 = 34.0; 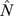 = 16,075.

From settlement and environmental factors we turn to vital rate parameters, the first of which is density independent, background mortality, implemented as an increase in the baseline expected lifespan. Increasing background mortality causes a pronounced increase in the length of the copial phase (File S1 Table S1d). In an environment in which even a well-fed individual (*E*≥1) lives only 30 years on average, the effective growth rate is so small that it takes 1204 years to fill the habitat to *E* = 1 (Figure 7). If that individual expects to live to 45 or even 60 years on average, the population grows rapidly enough that it reaches *E* = 1 within 347 or 237 years, respectively. Similarly, as background mortality declines, so does *MTI*. Populations in environments we would consider propitious will, once they experience hunger, approach their Malthusian destiny more rapidly, with less time to react. *MTI* = 65 years for a baseline expected lifespan of *e*_0_ = 30 and drops to 48 years for baseline *e*_0_ = 60.

Expected lifespan is the first parameter we have examined for which the equilibrium values for birth and death rate and, as a consequence, other demographic quality of life variables differ among treatments. When background mortality is high, the equilibrium population reaches a density of 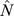 = 10,441, with an equilibrium life expectancy of 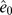 = 26.8 years, and a relatively low level of hunger, consuming 85.1% of its baseline kilocalorie diet. By contrast, when background mortality is low, the equilibrium population is much larger, 16,075. While it has a longer equilibrium expected lifespan (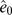 = 34.0), it suffers hunger at the alarming level of 56.2% of its baseline diet. High background mortality results in a smaller population with shortened expected lifespans but less hunger at equilibrium for those individuals who survive their otherwise harsh conditions.

These results demonstrate that equilibrium conditions are set by the combination of density independent and density dependent mortality, offsetting fertility. As density independent mortality increases it takes less food-induced impedance to sustain the Malthusian equilibrium. Lessened background mortality generates a population abundant in numbers but poorly fed. Population control by strictly Malthusian means is a punishing situation either way.

#### Fertility

**Figure 8:**
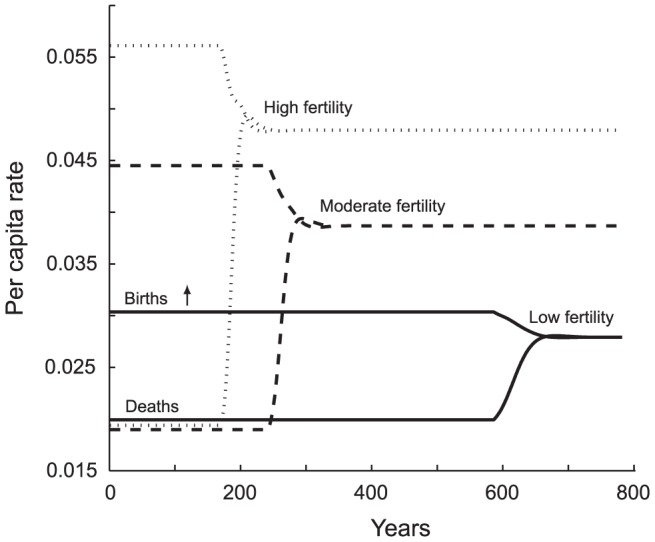
Effect of Total Fertility Rate (*TFR_d_*) on the Time Course of Vital Rates. Assessing *TFR_d_* at *E* = 1, the low-fertility population's *TFR_d_* is 2 daughters per mother (solid line), the moderate *TFR_d_* is 3 (dashed line) and the high fertility population has a *TFR_d_* of 4 (dotted line). Mortality tends to be more responsive than fertility in our parameterization of the model, so when fertility is high the gap between the vital rates is closed at equilibrium by a marked rise in morality. The result is higher fertility *and* mortality at equilibrium than in the other scenarios. Generally, lower maximum fertility also means lower per capita mortality at equilibrium, as shown by the per capita rate at which morality and fertility converge. The high-fertility population also grows quickly; it thus has a shorter copial phase, experiences hunger sooner, and has a steeper, more abrupt approach to equilibrium. It is the least well-fed at equilibrium and has a very short life expectancy. In numbers: High fertility population: 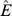 = 0.579; 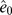 = 20.4; 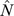 = 15,956. Moderate fertility population: 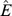 = 0.624; 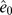 = 25.4; 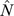 = 14,601. Low fertility population: 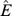 = 0.731; 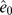 = 35.3; 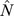 = 12,247.

The effect of different per capita fertility rates mirrors that of death rates, an expected result in that *r* = *d* – *b* (Figure 8; File S1 Table S1e). Higher total fertility (*TFR_d_*) results in a sharp decline in the duration of the copial phase and a less evident decline in the much shorter, transitional phase. High fertility populations approach the point of hunger quickly; they likewise move from *E* = 1 toward equilibrium at a faster rate than a population with lower baseline fertility.

A low initial fertility rate, set at a *TFR_d_* of 2 daughters per woman, corresponds to greater food availability at equilibrium (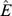 = 0.731), a longer average life span (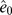 = 35.3), and a smaller population of 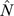 = 12,247 (Figure 8). Malthusian equilibrium is achieved by a relatively small increase in density dependent, per capita death rates (0.0199 to 0.0279) matched to a lesser drop in birth rates (0.0303 to 0.0279). By contrast, for the high fertility population (*TFR_d_* = 4), the Malthusian state is one of greater hunger (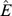 = 0.579), shorter expected life span (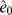 = 20.4) and a larger population (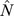 = 15,956). Per capita death rate at equilibrium has increased by a substantial 2.5 times, from 0.0194 to 0.0479, again reflecting the brutal mechanics of Malthusian controls.

#### Elasticities of Survival and Fertility

**Figure 9:**
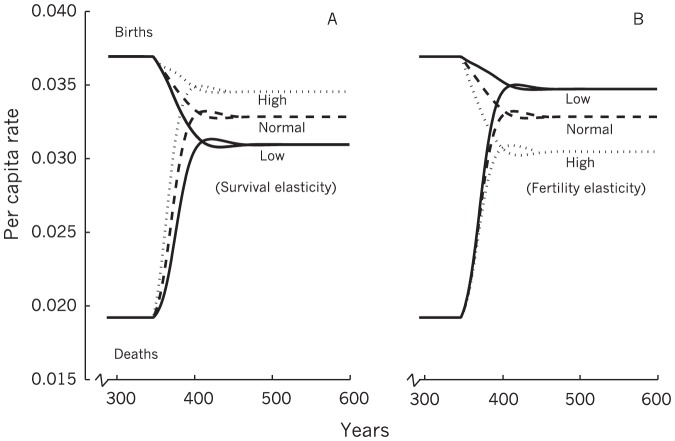
Effect of Elasticity on the Time Course of Vital Rates. (A) Elasticity of survival (mortality); (B) Elasticity of fertility. Elasticities play no role in the duration of the copial phase (much of which is elided in the graphs); they increase sensitivity of vital rates to hunger, shortening the duration of the transitional phase. Increasing the elasticity of mortality at *E* = 1 from half its empirical value (labeled “low” in the graphs) to its empirical value (normal) to double its empirical value (high) causes mortality to climb more quickly once *E* drops below 1, resulting in relatively high equilibration of per capita birth and death rates. Higher elasticity of fertility at *E* = 1 causes birth rates to drop more quickly with births and deaths. High elasticities are associated at equilibrium with more adequate Kcal intake, longer lifespan and lower population size. In numbers: Low survival elasticity: 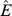 = 0.622; 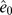 = 31.8; 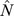 = 14,530. Normal survival elasticity: 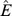 = 0.668; 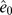 = 30.0; 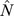 = 13,509. High survival elasticity: 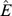 = 0.721; 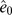 = 28.5; 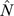 = 12,478. Low fertility elasticity: 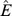 = 0.652; 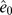 = 28.3; 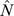 = 13,894. Normal fertility elasticity: 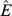 = 0.668; 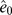 = 30.0; 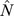 = 13,509. High fertility elasticity: 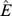 = 0.694; 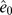 = 32.3; 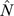 = 12,959.

Finally, we consider the responsiveness of age-specific vital rates to shortfalls in food availability (Figure 9; File S1 Table S1f). Because density dependence operates only if *E*<1, the elasticity of vital rates has no effect on the duration of the copial phase. However, increasing the elasticity of vital rates to food shortfalls shortens the transition phase (File S1 Table S1f). We portray this result by multiplying elasticities in the vicinity of *E* = 1 by values from 0.5 to 2, a procedure that captures increasing sensitivity to a unit decline in food availability. Vital rate elasticity can be conceptualized as determining which of the per capita vital rates, death or birth, converges most quickly on the other and, consequently, which makes the greatest contribution to population equilibrium. Keeping in mind that these rates are falling, greater elasticity of survival rates enhances the impact of mortality (per capita death rate) and greater elasticity of fertility rates enhances the impact of fertility (Figure 9A, 9B) on the achieved equilibrium. For both parameters, this entails a more rapid transition to Malthusian equilibrium. Note, however, that the 4-fold change in vital rate elasticities depicted in Figure 9 has a fairly modest impact on equilibrium values for the food ratio (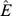), life expectancy (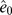), and population size (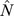).

## Discussion

### Resource-limited Population Phases and Their Determinants

Naturalistic population growth in a space-limited agrarian population is characterized by copial, transitional and Malthusian phases. We introduced the term *Malthusian transition interval (MTI)* to characterize the brevity of the transition phase and its singular consequence: a rapid shift from full diet and positive demographic indicators to hunger and depressed demographic indicators (Figure 3). Malthusian constraints arrive abruptly, with little warning; once initiated, they settle onto a population rapidly. Their impact on quality of life variables can be quite severe; *ceteris paribus*, they last indefinitely. Ironically, the better the copial phase conditions that precede transition to Malthusian equilibrium – e.g., high fertility, low background mortality (longer lifespan) – the quicker and grimmer will be the plunge to the Malthusian condition when it arrives. When the pressure on basic demographic adjustment is greatest, the time available to respond adaptively is shortest.

Passage times through the copial and transitional phases depend on environmental and demographic parameters (File S1 Table S1). Increased size of the founding group *N_t_*_ = 0_ shortens the copial phase; increased area available for settlement *A_m_* lengthens it. In both cases the relationship is log-linear. Neither variable, however, affects the duration of the transition phase. Yield (*Y*), has opposing effects. Very low yield may eliminate the copial phase altogether; increasing yield lengthens the copial phase but it shortens the transitional phase. Longer baseline expected lifespan (*e*_0_) shortens both the copial and transitional phases, the former to a much greater degree than the latter. Greater baseline fertility (*TFR_d_*) has the same effect. Finally, increasing the elasticity of fertility and mortality in response to food shortfalls has no effect on the copial phase, but shortens the transition phase.

In all circumstances except that of very low yield (*Y*), the duration of the copial phase exceeds that of the transition phase by a wide margin. Realistic gamma function parameters for the elasticity of survival and fertility produce another result: for all of our variables and scenarios, increasing mortality contributes more to the convergence of vital rates and thus to equilibrium than does declining fertility (Figures 4 through 9). This is a direct reflection of the fact that elasticities of survival are larger than those of fertility near *E* = 1 [11].

### Resource-Limited Population Ecology

Most previous attempts to define population growth rates in terms of resources either lack demographic complexity or are not developed into full dynamic models (e.g., [17],[19],[20],[21],[22]). We seek more complex dynamics from the bottom up, by defining explicit links among environment, labor capacity, caloric requirements, competition, food availability and vital rates (Figure 1). Other models assume that there is a single “density dependent” vital rate (e.g., [23]), that vital rates respond in sequence from most to least sensitive [24], or that the population is made up of a single age class and the overall growth rate is the food-dependent variable (e.g., [22]). Our framework attains greater realism through the use of elasticities to quantify a proportional response of age-specific vital rates to food deficit.

In general, the literature on the population ecology of pre-modern societies takes as its inspiration the logistic model of Lotka-Verhulst [6]. The logistic calculates impedance to an underlying exponential growth rate, assuming that the growth rate slows from the very moment that population increase begins. The factor damping growth is either linear, as in Verhulst's original formulation, or curved, as in the theta logistic [25]. Equilibrium occurs when the population arrives at a carrying capacity assigned *a priori*. This pattern of impedance and point of carrying capacity are either deduced from first principles or derived statistically from fitting the model to empirical observations [26].

We address two shortcomings of the Lotka-Verhulst approach. First, because the impact of the Lotka-Verhulst feedback term is gradual and is felt from the very beginning of growth, it necessarily entails a smooth approach to equilibrium over the whole course of a population's trajectory. There is nothing like a copial phase of unchanging maximal growth, no possibility of a prolonged surplus, and no sharp discontinuities in growth rates (cf. Figures 2, 3), in short, no abruptness to the dynamics. The Lotka-Verhulst approach produces dynamic gradualism; our model allows for a more punctuated form of population history.

The second shortcoming is related. In the Lotka-Verhulst model, both *r* and carrying capacity (typically, *K*) are set as initial parameters. In our approach, these two variables are outcomes of lower-level variables, some of them easier to observe and assess empirically. This potentially increases the empirical reliability and the analytical flexibility of the model. Instead of a single value for the intrinsic rate of increase, *r*, we are able to derive growth from birth and death rates over age structure, represented as elastic responses to food intake. Instead of a single measure of carrying capacity, we build up to density dependent resource limitations from estimates of agricultural area, yield, age-specific work effort, and interference competition.

### Social Evolution and the Question of State Collapse

Hypotheses that population has had a causal role in social evolution are prominent in both the popular [27] and the scientific literature [28],[29],[30]. Population pressure is seen to compel new adaptive forms like agricultural production or state-level political organization, or it is seen to exceed the adaptive capacity of societies and lead to their decline or collapse due to environmental degradation or peasant revolt. Population competes with other explanatory candidates, among them environmental change, economic innovation, warfare, ideological development and the agency of individuals seeking prestige. Unifying frameworks are rare but needed, given the complexity of human society and the many factors that reasonably have been postulated to cause early civilizations to rise, persist and fail. By including in a single systematic framework, environment, labor productivity, nutritional requirements, age structure and vital rate demography our approach seeks to provide a more synthetic framework.

Interactions among population, labor and food availability are often implicated in collapse narratives, but the models on which these arguments are based may not be sufficient to the job. We focus here on the Maya case, because it is one of the best known cases in which advanced agrarian political systems failed. For example, Culbert [31] proposes that Classic Maya political society collapsed because of a labor shortage. Although approaching high levels of population, Maya society was vulnerable to diversion of workers from agriculture to warfare and the construction of monumental architecture. By contrast, a population ecology approach suggests that when the population is large and has limited ability to expand into agricultural frontiers there is no better time to divert workers from the fields, as their marginal productivity in agriculture is approaching zero. If relatively few workers were needed to construct the monuments and other public works of Maya polities [32], there would remain a pool available for diversion into warfare without much impact on agricultural production. Redirecting farmers to become soldiers who suffer elevated (density independent) mortality might actually *stabilize* subsistence production, as the non-warrior, producing population would suffer less hunger and enjoy a longer expected lifespan. Empirically, Culbert [31] might be shown to be correct, but counter-intuitive elements of resource-limited population ecology reduce the *a priori* plausibility of the proposed hypothesis.

Some analysts of Maya prehistory [33] propose that the agrarian workforce caused the collapse of Maya polities by revolting against intolerable levels of exploitation. Population ecology insights can elevate or diminish the likelihood of such a hypothesis being correct. For instance, its plausibility is low if producers are in the copial phase of population growth, but greater if they already are suffering from the effects of a Malthusian equilibrium. High density independent background mortality would lower the plausibility of this hypothesis by elevating levels of food availability at equilibrium; low background mortality would make it more likely.

A central question in the collapse literature is whether we need look exclusively to exogenous factors for the causes of collapse. Societies have been challenged from the outside: Huns attack, droughts and pestilence ruin crops, foreigners introduce new diseases. However, endogenous practices also can contribute to sudden instability (Figure 3). Social practices affecting work, fertility and consumption, can result in severe dislocations, depending on where in its growth trajectory a population happens to be. Innocuous practices such as high fertility established early in a growth cycle, in times of plenty, may become maladaptive later in that cycle.

Turchin [34] finds evidence that agrarian societies exhibit low amplitude population cycles on a period of 200–300 years, a wave-length roughly consistent with our observation of growth to equilibrium over 6–12 generations. Turchin proposes a “demographic-structural” theory that envisions rapid population growth provoking the breakdown of central state authority, which in turn generates conditions (e.g., lawlessness, conflict, degradation of public infrastructure protecting health and food production) that eventually cause a population downturn. Lowered population levels are then followed by restoration of public order and investment, and a renewed cycle of growth. Temporal lags between the linked cycles of population and public order drive the out-of-phase historical observations of oscillations. Our emphasis on the punctuated character of Malthusian constraints is consistent with the demographic-structural approach, but would place greater weight on the structural impact of environmental and demographic variables. Even if central authority remains intact and the more violent expressions of public breakdown muted, it may prove difficult for a society to avoid a prolonged demographic downturn when faced with abrupt imposition of food-limited, Malthusian constraints.

Our reading of the state collapse literature suggests that there is an overwhelming preference for hypotheses based on external, often environmental causation [29],[35]. Extreme drought [36],[37],[38],[39],[40],[41] is a favorite candidate. Population, if considered, is in the background, and is seen as a gradual process raising vulnerability to a sudden but exogenous insult. We see in this view the lingering influence of Lotka-Verhulst and the association of density dependent population growth with gradualism. But population ecology processes that are gradual in some respects (e.g., declining surplus, increasing density), can entail abrupt and dramatic changes in others: average life span, fertility and survival among them (Figure 3). As quality of life indicators these demographic variables measure potential for social disruption. An unexpected plunge over two generations from abundance to the grim reality of Malthusian limits acts with nearly the same suddenness and degree of impact as a short sequence of recurring droughts. A demographic crisis may be just as unexpected, impose levels of hunger (*E*) and hardship just as severe. *MTI*, the opportunity to forestall by adjustment, will be just as short.

Fortunately, we can predict how particular environmental or demographic circumstances will affect the timing and magnitude of punctuated demographic change. A large habitat area extends the period of copial abundance experienced by a founding population, but it does not prolong the descent to equilibrium once it starts. High yield likewise delays the eventual Malthusian reckoning, but it then shortens *MTI*. A low-yield environment is the only circumstance among the scenarios that we examine in which demographic variables signal an impending equilibrium gradually over the course of habitat in-fill (Figure 6). Demographically hospitable environments, measured by low background mortality and high fertility, shorten both the copial and transitional phases. As measured by hunger (*E*<1), they impose especially severe food shortages once the Malthusian phase occurs. Across all environments, high population density is achieved at a high cost to quality of life and, across most all environments, low yield again being the exception, *MTI* is quite short relative to the length of the food-abundant phase. Each of these predictions tells us something about the likelihood that rapid demographic change lies behind population upheaval and socio-political collapse in particular cases.

An agrarian producing population may endure at a high density set by food-limited constraints even as the rapid decline in human welfare associated with the Malthusian transition phase causes, or abets the causes of, political collapse. This is consistent with current thinking in the Maya case, which entails the interaction of high population density, increasing warfare, and a gradual and long-standing environmental downturn [42]. Malthusian disruption to the social fabric and infrastructure of everyday life might well be enough to drive a highly interconnected civilization to fail, whether or not suffering other stresses.

### Bioarchaeology and Population History

Anthropologists commonly use bioarchaeological assessments of human health and nutritional status from mortuary populations to assess whether or not prehistoric societies are suffering from food shortages. This methodology and interpretations resulting from it are then used to assess the likelihood that over-population or habitat degradation explain socio-political developments. Absent evidence of osteological insult during the lead-up to a phenomenon like social collapse, population-based explanations are considered to be refuted. For example, in their survey of bioarchaeological evidence from Maya prehistory, Wright and White [43]:147 state that the ecological model for lowland collapse and abandonment “requires … consistent change in diet from Preclassic and Early Classic Periods to the Terminal Classic.” This imposes a requirement of gradual increase in measures of nutritional deficit over 600–800 years of expanding population density. Wright and White find instead that the isotopic and bioarchaeological evidence shows local diversity and no trends through this lengthy time span. They interpret this result as inconsistent with ecological or population imbalance models.

However, underlying this interpretation is the assumption that the stress of over-population must build gradually over a significant period of time prior to a dramatic event like socio-political collapse. This assumption would be consistent with a Lotka-Verhulst approach; it is not consistent with our model results showing a long period of relative abundance that ends abruptly with a rapid transition to Malthusian straits. Given the standard error ranges of archaeological dating, and the aggregate nature of mortuary populations, in all except the very low-yield cases evidence of nutritional shortfalls will appear in the archaeological record more-or-less coincident with their social consequences (e.g., collapse). They may be dismissed as an effect rather than a cause. Surplus will decline slowly in the long-term lead-up to disruptive demographic stress (Figure 3) – the granaries slowly will go empty and the rats become skinny and sparse – but in a growth trajectory we should not expect to find bioarchaeological indicators of stress in humans until the Malthusian crisis of density dependence is only a few generations from its full and crushing impact.

## Conclusion

Societies are complex systems linking natural and social features [44]; their study requires models that do the same, while drawing on comparative, historical and prehistorical records [45]. The modeling effort we describe instantiates these goals in a population ecology approach that embodies the fundamental principles discussed by Turchin [46]: exponential growth, self-limitation and consumer-resource oscillation. With Turchin [47], we take population ecology models to be tools for seeking understanding of regularities in history.

Colonizing populations or those recovering from an epidemic or other catastrophe so that they are faced with comeback in a relatively empty habitat will enjoy a lengthy period of comfortable growth before they hit a quick transition to deprivation of indefinite length. Except for a very brief transition, populations in which fertility and mortality are density dependent will exist in one of two states: comfortable or deprived. This runs counter to intuitions formed by familiarity with variants of the logistic equation.

We propose that growing populations in the copial phase have few means of detecting their fate more than several generations in advance of it being fully upon them. It lurks like an invisible cliff. Their *Malthusian transition interval (MTI)* is short. From initial settlement, the growth rate of colonizers remains constantly high and population size increases at an accelerating rate. Justifications for complacency abound. Surplus food availability declines but until food availability drops below the critical value of *E* = 1, quality of life variables like life span, infant survival and fertility are unaffected by habitat in-fill. Absent nutritional or demographic strain felt at the household level, swelling aggregate population size is unlikely to be perceived or monitored as a signal of pending trouble. Similarly, we do not expect individuals to anticipate crisis from a declining surplus; food availability in excess of need weakens the signal. Growth rate (*r*) by contrast reflects palpable and personal linkages among mortality, fertility and hunger, and it or its constituent vital rates should constitute a strong household signal of environmental difficulty. However, these are constant until a few generations before the Malthusian equilibrium. The most informative and directly reliable signals, those entailing hunger and acute changes in mortality and fertility, offer virtually no lead-time.

The brevity of the *MTI* limits options for response if adaptations require a long lead time or heavy investments of planning and implementation. It is difficult to envision that a society could successfully undertake the introduction of facilities like extensive irrigation canals, terracing or raised fields on the timespan of a couple of generations after perceiving their need, during a crisis. In a sanguine conclusion to his review of the “Archaeology of Overshoot and Collapse,” Tainter [28]:72 says:

“overshoot is teleological, as if humans could set a target for population or consumption…[it] denies the human capacity for flexible adjustments…It usually is possible to coax more resource production by applying capital and technology, increasing labor, applying energy subsidies, and making production more knowledge-intensive.”

Given realistic appraisal of how unexpectedly and quickly they may impose themselves, population crises may offer little opportunity for the kind of adjustments Tainter envisions.

These observations also suggest, however, that we should be cautious in extrapolating from the fate of places like Easter Island to our own situation [48]. The collapse of Pacific Island societies sometimes is portrayed as a failure to recognize the need for adaptive response. Plentiful signs were not read or were ignored, reasonable adjustments were not made, and social upheaval ensued when population finally exceeded resource capacities (cf. [49]). But this reading of the evidence entails a Lotka-Verhulst version of the world in which human failures are measured by long-standing neglect of distress signals. Our results suggest that the very salubrious Pacific Island setting is precisely the one in which the signal would anticipate problems by the slimmest of margins, perhaps coming too late for an effective response no matter how conscientious the population.

Many models that place population at the heart of social upheaval envision linked resource degradation as the ultimate cause. Overexploitation provokes deteriorating yields or, in the Maya case “a population collapse precipitated by agricultural failure” [50]:141. This combination of causes may well have been the case historically. But it is important to acknowledge that population can act abruptly and with severity even in the absence of an environmental crisis. In our analyses the environmental capacity to yield resources is not declining; our plunge to Malthusian constraints does not invoke factors like reduced yield, forest clearance or soil erosion. Rather, the fate of the population resides primarily in the responses of fertility and mortality in the face of calorie shortages. Population stress and collapse might arise from punctuated demographic failure quite apart from environmental manifestation of resource overexploitation. Abrupt imposition of Malthusian constraints and insufficient *MTI* may well be a stand-alone culprit in social evolution.

## Supporting Information

File S1 **Three sections of supporting material, including Table S1 and two sections of text.**
(DOC)
