# Supplementary material for: The Invisible Cliff: Abrupt Imposition of Malthusian Equilibrium in a Natural-Fertility, Agrarian Society
Source: PLoS One. 2014 Jan 31;9(1):e87541. doi: 10.1371/journal.pone.0087541 (PMC3909123; doi:10.1371/journal.pone.0087541)
Supplement: File S1 — Three sections of supporting material, including Table S1 and two sections of text. (DOC) [file pone.0087541.s001.doc]

# File S1, Section 1.

Table S1. Copial and transitional phase duration as a function of basic parameter values.

| **Increasing** | **Causes the copial phase to be** | **And the transition phase to be** | **Graphs** |  |
| --- | --- | --- | --- | --- |
| (a) Size of founding population (*N*_0_) | Shorter | Unchanged |  |
| (b) Area available for habitation (*A_m_)* | Longer | Unchanged |  |
| (c) Yield (*Y*) | Longer | Shorter |  |
| (d) Baseline life expectancy | Shorter | Shorter |  |
| (e) Baseline fertility | Shorter | Shorter |  | |
| (f) Elasticity of fertility & mortality | Unchanged | Shorter |  | |

# File S1, Section 2: Passage Time, Narrative

Ideally we would like to predict the duration of the copial and transitional phases directly as a function of input parameters (Figure 1). We could then determine the duration of each phase under any combination of the environmental and demographic conditions that the model can represent. The complexity of our model forestalls simple analytical ways of doing this. Instead, we rely on approximations. These allow us to confirm from equations the structural properties we observe through iteration (File S1 Table S1), and to ascertain the accuracy of quantitative estimates. Our primary objective is to estimate the time required to get from one point to another on a population’s growth trajectory, focusing on our phase boundaries.

## Length of copial phase

We obtain from File S1 Section 3 (S3.8) the following, second-order Taylor expansion estimate for the time to pass from founding to *E* = 1:

where *E_m_* is the density independent maximum value of the food ratio, *E*, as *N* approaches 0 [12] and *B* is or a measure of the level of interference competition among workers when the population is founded. A larger *B* means low competition, which allows new land to be put into use faster as the population grows.

This estimate shows us the bases for the structural properties observed in copial phase duration (File S1 Table S1); accurate quantitative predictions require a less transparent and cumbersome fourth order Taylor expansion (see File S1 Section 3). The initial term (1/r_0_) indicates that copial phase duration will decline asymptotically as either the birth rate increases (File S1 Table S1e) or mortality declines (File S1 Table S1d). The term 2/*B* increases with increasing area (*A_m_*) and shrinks with a larger initial population size (*N*_0_). The copial phase lengthens as this term increases and shortens as it decreases, in log-linear fashion. The second fraction within the log term captures how far *E* can fall within the copial phase from its theoretical maximum, as a fraction of that maximum. The richer the environment and the greater the productivity of workers, the greater this fraction becomes. The result of an increase is a log-linear increase in the length of the copial phase. However, if *Y* is small enough that *E_m_* ≤ 1, meaning that the maximum value of *E* is insufficient to exceed the ideal vital rate needs of the smallest population, then the log term will be non-positive and there is no solution, in effect, no copial phase (see File S1 Table S1c; values of *Y* at or below 6,747 kcal/ha/day).

## Length of transition phase

To calculate transition phase length we integrate from the population’s size at *E* = 1 to its size at the boundary with the Malthusian phase, set at a doubling time = 1000 years (S3.19). (S3.19) cannot be solved analytically; it requires numerical integration. Despite the number of terms, (S3.19) boils down to an estimate of the area under 1/(*r N*) as a function of *N*.

Neither initial population size (*N*_0_) nor area (*A_m_*) has any effect on *MTI* (Table 2a, 2b). Wherever population and area terms appear in (S3.18), they do so as a density inverse (*A_m_*/*N*_0_), which corresponds to a unique *E_t_* along the growth trajectory. Note that *g* stands for population size within the integral in (S3.18). The copial phase length changes in response to *N*_0_ and *A_m_* because these represent different starting points on the single path of *E* as a function of density. However, neither of these two parameters affect the transition phase because, regardless of the initial conditions *all* populations in the same environmental and demographic context travel the same density dependent path between *E* = 1 and *Ê*. Initial conditions of population size and agricultural area cease to matter once *E* = 1, assuming that the population is not already in the transition phase at *t* = 0.

Yield (*Y*) is unusual in that its effect on the transition phase is the opposite its effect on the copial phase (File S1 Table S1c). Greater yield shortens *MTI*. The reason is that the population reaches *E* = 1 at larger size and thus with the momentum to pass more quickly through the transition to equilibrium. The effects of the demographic parameters on *MTI* can be understood by their effect on the growth rate. Simplifying, $t\approx\int\frac{1}{rN}\delta N$, and thus parameters that increase *r* will shorten the transition phase (S1d, S1e). Unlike in the copial phase, elasticities of vital rate response to food limitation also play an important role in the transition phase. Greater vital rate elasticity as a function of *E* means a more rapid convergence of birth and death rates (Figure 9; File S1 Table S1f) and a shorter transition.

# File S1, Section 3: Passage Time, Mathematical Approximations

## The Copial Phase

In a space-limited agricultural system without adaptation, and assuming that no initial investment of labor is needed to reach agricultural potential, we assume a small initial population arrives and is capable of producing a surplus. While food production is sufficient to meet the population’s dietary needs the population grows at is maximum rate, *r*_0_. We call this the ‘copial’ phase of growth; *E* ≥ 1. If we assume the population has stable age structure **u**_0_, corresponding to *r*_0_, the population vector at time *t* can be written **n***_t_* = *N_t_* **u**_0_, where *N* is a scalar representing the population number.

Define

$A=\frac{YA_{m}}{J\rho_{0}N_{0}}$ (S3.1)

(S3.2)

where the space-limited model parameters represent their usual selves, with the addition that and . The angle brackets refer to the scalar product of two vectors. We are interested in the time it takes the population to grow from initial size *N*_0_ to the point at which *E* =1, whose population we will call *N_E_*_=1_:

(S3.3)

We can determine *E* at any point from our food ratio equation. If we assume structure **u**_0_ and the usual exponential form of *F* [see 12],

(S3.4)

When *E_t_* = 1, *N_t_* = *N_E_*_=1_ and,

(S3.5)

A second-order Taylor expansion of allows (S3.5) be approximated analytically for *t* when *E* = 1,

(S3.6)

(S3.7)

, (S3.8)

where , which is the theoretical maximum value of *E*, occurring when the population is infinitesimally small [12].

As an expression of the parameters,

(S3.9)

When baseline *e*_0_ = 45, *Y* = 21,000 Kcal/ha/day and we use our typical

parameters for the rest, the second-order approximation is passable (suggesting it takes 20 colonists 300 years to get to *E* = 1 versus the actual 347 years). A fourth-order approximation is more accurate (323 years), but forsakes transparency:

(S3.10)

## The Transitional Phase

Working from the definition for the food ratio, as before, we can now solve for how long it takes to get from *E* = 1 to the neighborhood of $\hat{E}$, the so-called Malthusian phase in which vital rates are diminished due to the effects of inadequate food supply and the population growth rate declines as a result.

From the exponential growth equation we can express population size at time *t* in terms of *N*_0_ and the mean exponential growth rate when *E* < 1. The mean growth rate when food is less than adequate is called $\bar{r}$,

, (S3.11)

where *N*_1_ is the population when *E* = 1 and *t*_2_ is the time between *E* = 1 and a value of *E* less than 1.

We solve for *E_t_*, using the previous expression for population size in (S3.4), then perform a second-order Taylor expansion on the exponent in the numerator,

(S3.12)

Solving for _t2_ gives,

(S3.13)

where *E_Q_* is the target value of *E*, corresponding to *t*_1_ + *t*_2_. This approach is of limited utility as we have so far been unable to approximate $\bar{r}$ as a function of *t*. However, from the appendix of Puleston and Tuljapurkar [12] (see Section A.5) we have an excellent approximation of *r* as a function of *E*, which may be rewritten,

, (S3.14)

where *K* is a constant that describes how the elasticities of the vital rates change with *E*, *T* is the generation time and $\xi^{0}$ is a constant that describes the mean of the baseline elasticities. This is an expansion around the point where *E* = 1, but with *K* = 7.4, T = 27.9 and $\xi^{0}$ = 0.327 the approximation of *r* is quite good until very close to $\hat{E}$. We may substitute (S3.4) into this equation to get *r* as a function of *N*.

## Malthusian transition interval (MTI)

From our understanding of exponential population growth,

(S3.15)

Rearranging we solve for *dt*,

(S3.16)

Integrating gives,

(S3.17)

We convert (S3.14) to express *r* in terms of *N*, then substitute into the integral, giving an expression for *MTI*, or the time it takes to get from *N* = *N_a_* to *N_b_*,

(S3.18)

We assume *N_a_* ≤ *N_t_*_=1_ (meaning not in the copial phase) and *N_b_* ≤ $\hat{N}$, meaning that the expression approximates the system from any point when *E* ≤ 1 to its equilibrium. We assume the copial age structure (in *φ*_0_ and *ρ*_0_) because although the population is in transition between the copial and equilibrium structure, the transition occurs so quickly that the copial is an appropriate approximation. We can simplify the expression by observing that , meaning that these terms are just an expression of the food ratio at some population level *g*:

(S3.19)

It should be noted that because the age structure is changing during the transition phase the population tends to overshoot the equilibrium value and then settle back down to it. Simulation finds that the *N_b_* coincident with a doubling time of 1000 years exceeds $\hat{N}$, so our integral has no solution in this case. We have several options at this point. First, we can determine *N_b_* from a population that is constrained to maintain the copial age structure. This is equivalent to compressing the population into a single representative age class. Second, we might call the end of the transition stage at an earlier point, for example a doubling time of 200 or 500 years, to avoid falling into the region of the overshoot. Another option is to define the end of the transition phase in terms of *N_t_*. For example we might declare that when the population has covered 99% of the distance between *N_1_* and $\hat{N}$ that the transition is effectively complete and that the population is in the neighborhood of equilibrium.

Using the first method (constrained age structure to estimate *N_b_*) the approximation predicts 29 years to get from *N_t_* = 8641 to 12,500, the point when doubling time of the unstructured population has reached 1000 years. The observed span is 51 years, from the full simulation. On the other hand if we define the end of the transition phase at a doubling time of 200 or 500 years the approximation predicts 43.6 years and 62.7 years vs. observed spans of 38 and 47, respectively. Using the third method we ask how long it takes to get from 8641 to 13,460 (covering 99% of the growth required to get from the end of the copial to the equilibrium point). The approximation predicts 59 years and the full simulation requires 43. The approximation underestimates the rate at which *r* is falling as it gets very close to zero. This causes the error to blow up as we move the cutoff of the transition phase closer to the true equilibrium point.
